# Comparison of intravenous immunoglobulin and plasma exchange in treatment of mechanically ventilated children with Guillain Barré syndrome: a randomized study

**DOI:** 10.1186/cc10305

**Published:** 2011-07-11

**Authors:** Mohammed A El-Bayoumi, Ahmed M El-Refaey, Alaa M Abdelkader, Mohamed MA El-Assmy, Angi A Alwakeel, Hanem M El-Tahan

**Affiliations:** 1 Pediatric Intensive Care Unit, Mansoura University Children Hospital, Al-Gomhuria Street, Mansoura 35516, Egypt; 2 Pediatric Nephrology Unit, Mansoura University Children Hospital, Al-Gomhuria Street, Mansoura 35516, Egypt

## Abstract

**Introduction:**

Respiratory failure is a life threatening complication of Guillain Barré syndrome (GBS). There is no consensus on the specific treatment for this subset of children with GBS.

**Methods:**

This was a prospective randomized study to compare the outcome of intravenous immunoglobulin (IVIG) and plasma exchange (PE) treatment in children with GBS requiring mechanical ventilation. Forty-one children with GBS requiring endotracheal mechanical ventilation (MV) within 14 days from disease onset were included. The ages of the children ranged from 49 to 143 months.

Randomly, 20 children received a five-day course of IVIG (0.4 g/kg/day) and 21 children received a five-day course of one volume PE daily. Lumbar puncture (LP) was performed in 36 patients (18 in each group).

**Results:**

Both groups had comparable age (*p *= 0.764), weight (*p *= 0.764), duration of illness prior to MV (*p *= 0.854), preceding diarrhea (*p *= 0.751), cranial nerve involvement (*p *= 0.756), muscle power using Medical Research Council (MRC) sum score (*p *= 0.266) and cerebrospinal fluid (CSF) protein (*p *= 0.606).

Children in the PE group had a shorter period of MV (median 11 days, IQR 11.0 to 13.0) compared to IVIG group (median 13 days, IQR 11.3 to 14.5) with *p *= 0.037.

Those in the PE group had a tendency for a shorter Pediatric Intensive Care Unit (PICU) stay (*p *= 0.094).

A total of 20/21 (95.2%) and 18/20 (90%) children in the PE and IVIG groups respectively could walk unaided within four weeks after PICU discharge (*p *= 0.606).

There was a negative correlation between CSF protein and duration of mechanical ventilation in the PE group (*p *= 0.037), but not in the IVIG group (*p *= 0.132).

**Conclusions:**

In children with GBS requiring MV, PE is superior to IVIG regarding the duration of MV but not PICU stay or the short term neurological outcome.

The negative correlation between CSF protein values and duration of MV in PE group requires further evaluation of its clinical usefulness.

**Trial Registration:**

Clinicaltrials.gov Identifier NCT01306578

## Introduction

Guillain-Barré syndrome (GBS) is, currently, the most common cause of acute flaccid paralysis following the worldwide decline in incidence of poliomyelitis. Incidence varies according to age, geographic areas and diagnostic criteria used for inclusion. Annual incidence in western countries varies from 1.1 to 1.8/100,000 population per year [1-5] with a considerably lower annual incidence of 0.66/100,000 population per year in each of Taiwan [6] and China [7].

GBS usually follows infection by a number of bacterial and viral agents with *Campylobacter jejuni *representing the most common preceding infection [8-11]. The syndrome is also reported to rarely temporally follow vaccination with measles vaccine [12,13], tetanus toxoid [14], rabies vaccine [15], oral polio vaccine [16], polysaccharide meningococcal vaccine [17], measles-rubella vaccine [18], flu vaccine [19] and hepatitis B vaccine [19].

Since the publishing of the first report of the condition by Guillain, Barré, and Strohl in 1916, GBS has remained a clinically-diagnosed disorder. The condition is a polyneuropathy involving mainly motor but sometimes also sensory and autonomic nerves. It starts with rapidly progressive bilateral and relatively symmetric weakness in the lower limbs with diminished or absent deep tendon reflexes. Paralysis follows an ascending pattern involving trunk, upper limb and, finally, bulbar muscles. There can be numbness, parathesia and muscle pain and tenderness. Labile blood pressure with postural hypotension and labile heart rate with episodes of bradycardia up to asystole rarely occur denoting autonomic neuropathy. van Doorn *et al*. categorize diagnostic features of the condition into features required for diagnosis, including progressive weakness in both arms and legs, and areflexia or hyporeflexia, and features that strongly support the diagnosis, including progression of symptoms to a nadir over days to four weeks, relative symmetry of symptoms, mild sensory symptoms or signs, cranial nerve involvement, autonomic dysfunction, pain, a high concentration of protein in CSF without increase in cells and typical electro-diagnostic features [20].

A common, yet not an early, feature of GBS is increased cerebrospinal fluid (CSF) protein (> 45 mg/dL) without CSF pleocytosis (< 10 cells/mm^3^), often referred to as cytoalbuminous dissociation [21]. Electromyography may be used to confirm the diagnosis in the small subset of patients where the diagnosis is not straightforward. It is also useful to sub-classify patients into motor axonal neuropathy and acute inflammatory demyelinating polyneuropathy [22]. A recent study conducted in Egypt on children with GBS found that acute inflammatory demyelinating polyneuropathy, was the most common type (76%) while, acute motor axonal neuropathy, acute motor sensory axonal neuropathy and unclassified forms represented 8% each [23].

Respiratory failure is one of the most serious complications of GBS. It affects 15% of children with the condition [24]. The ability to predict the occurrence of respiratory failure and need for mechanical ventilation (MV) among patients with GBS has long been a target for neurologists and intensivists alike. Some bedside indicators of the likelihood of requiring MV are rapid disease progression, bulbar dysfunction, bilateral facial weakness, or dysautonomia, inability to stand, inability to lift the elbows or head, elevated liver enzymes and abnormal pulmonary function test [25-27]. Electrophysiological evidence of demyelination was also suggested to predict the need for endotracheal MV [28].

Treatment of GBS is a multidisciplinary effort. The general care of the child with muscle weakness includes regular monitoring of pulmonary and cardiovascular function for possible involvement of respiratory muscles and autonomic neuropathy respectively, prevention of infection and deep vein thrombosis, pain management, early physiotherapy, early rehabilitation once muscle power improvement starts and psychosocial support for the affected children and their families [20].

Specific treatment of severely affected children comprises intravenous immunoglobulin (IVIG) or plasma exchange (PE). Performed on more than 200 patients each, two large trials in the mid-1980s confirmed the beneficial role of PE in treatment of patients with GBS compared to the conventional treatment of the time [29,30]. The routine use of IVIG as the first line of treatment in GBS followed the publication of a randomized controlled trial (RCT) in 1992 showing a similar, if not a superior, effect of IVIG compared to PE [31]. A recent systematic review of heterogeneous study populations showed there was no evidence for a better outcome with either of the two modalities of treatment. There is also no evidence of a beneficial effect of adding corticosteroids to the treatment [32].

The objective of this study is to compare PE and IVIG as a first line treatment for children with severe GBS requiring MV regarding the duration of MV, PICU stay and short term neurological outcome.

## Materials and methods

Children with GBS admitted to the Pediatric Intensive Care Unit (PICU) at Mansoura University Children Hospital, Mansoura, Egypt, with the need for MV were prospectively enrolled in the study. Cases were diagnosed to have GBS according to clinical criteria by van Doorn *et al*. [20]. Patients were eligible for inclusion if they required MV based on the indications listed below, and if the duration of muscle weakness did not exceed 14 days on enrollment. Children were not eligible for inclusion if they had muscle weakness for longer than 14 days before requiring MV and if IVIG or PE was started prior to enrollment.

All patients were ventilated using endotracheal MV. Children were intubated if they were unable to protect their airway, had increased work of breathing (WOB), had PaO2 less than 70 mmHg in room air requiring increasing FiO_2_, or showed CO_2 _retention. When children were able to trigger spontaneous breathing, they were changed to a pressure-support spontaneous ventilation mode with continuous positive airway pressure of 5 cm H_2_O. Pressure support was gradually weaned to 10 cm H_2_O. If secretions were manageable and airway reflexes intact, a daily spontaneous breathing trial (SBT) was performed using a T-piece for two hours. Patients were extubated if SBT was successful. An SBT was deemed successful if there was no diaphoresis, increased WOB or apnea, tachycardia (defined by increase in the heart rate of 40 bpm or more) and if SpO2, pH, PaO_2 _and PaCO_2 _remained close to pre-SBT values. The decisions to initiate, wean and terminate MV were made independently by the attending consultant in accordance with and in strict adherence to the unit guidelines as above.

Over a period of three years, from January 2007 to December 2009, 44 children were admitted with GBS requiring MV; 41 children fulfilled inclusion criteria and were randomized to receive either IVIG (20 children) or PE (21 children) for initial treatment. Randomization was done by computer-generated random tables. IVIG was administered in a dose of 0.4 g/kg/day for five days. Patients in the PE group received a daily one-volume PE for five consecutive days. The sample size yields a study power of 86.9%.

On admission, the muscle power was recorded using the Medical Research Council (MRC) sum score [33]. Lumbar puncture (LP) was performed in the second week of illness in 36 out of the 41 children (18 in each group). LP was performed at the discretion of the attending consultant.

The primary outcome measure was the duration of mechanical ventilation and secondary outcome measures were length of PICU stay and ability to walk unaided within four weeks of PICU discharge. Institutional research ethics committee approval was granted and an informed consent was obtained from legal guardians of children included in the study.

Statistical analysis was performed using Statistical Package for the Social Sciences, Version 17 (SPSS, Inc., Chicago, IL, USA). *p-*value was considered significant if less than .05.

## Results

Children in the two treatment groups were comparable as regards base line characteristics, such as age, weight, duration of illness prior to requiring mechanical ventilation, presence or absence of preceding diarrhea, cranial nerve affection, severity of muscle weakness on PICU admission and CSF protein when available (Table 1).

**Table 1:** Comparison of IVIG and PE groups regarding base line variables

	IVIG group (*n *= 20)	PE group (*n *= 21)	*p-*value
Age (months)	106.0 ± 22.8 (74.3 to 113.5)	96.0 ± 32.8 (65.5 to 135.0)	0.764^1^
Weight (kg)	32.5 ± 7.0 (23.0 to 34.8)	29.0 ± 10.1 (20.0 to 41.5)	0.764^1^
DOI (days)	9.0 ± 2.7 (7.0 to 12.0)	9.0 ± 2.8 (6.5 to 11.5)	0.854^1^
Diarrhea (%)			
Yes	13 (65%)	12 (57.1%)	0.751^2^
No	7 (35%)	9 (42.9%)	
Cranial nerve affection (%)			
Yes	8 (40%)	10 (47.6%)	0.756^2^
No	12 (60%)	11 (52.4%)	
MRC sum score	12.0 ± 4.8 (8.5 to 15.5)	12.0 ± 5.9 (4.0 to 12.0)	0.266^1^
CSF protein (mg/dL)	152.1 ± 55.7 (88.3 to 173.6)	148.4 ± 43.0 (117.5 to 166.5)	0.606^1^

IVIG, intravenous immunoglobulin; PE, plasma exchange; DOI, Duration of illness prior to requiring mechanical ventilation; MRC, Medical Research Council score. Data presented as median ± SD (interquartile range)

^1 ^Mann Whitney test

^2 ^Chi square test.

There was a statistically insignificant better outcome in the PE group compared to the IVIG group. Favorable outcome was defined as the child's ability to walk independently for 10 meters (Grade 2 on GBS disability score) [34] within four weeks from PICU discharge. The post discharge management and physiotherapy were standardized for children in the two groups. In the IVIG group, favorable outcome was observed in 90% (18/20) of children compared to 95.2% (20/21) in the PE group (Table 2).

**Table 2:** Comparison of PICU stay and duration of mechanical ventilation in the IVIG and PE groups

	PICU stay (days)	Mechanical ventilation (Days)	Favorable outcome (%)	
			Yes	No
IVIG group	16.5 ± 2.1 (15.3 to 18.8)	13.0 ± 2.1 (11.3 to 14.5)	18 (90%)	2 (10%)
PE group	15.0 ± 2.6 (13.0 to 17.0)	11.0 ± 1.5 (11.0 to 13.0)	20 (95.2%)	1 (4.8%)
*p-*value	.094^1^	0.037^1^	0.606^2^	

IVIG, intravenous immunoglobulin; PE, plasma exchange. Data presented as median ± SD (interquartile range)

^1 ^Mann Whitney test

^2 ^Chi square test.

Children receiving PE had a significantly shorter period of mechanical ventilation compared to those receiving IVIG. The hospital stay was shorter in the PE group without statistical significance (Table 2).

For all patients, there was a statistically significant negative correlation between CSF protein and duration of mechanical ventilation (*p *= .024). Examining the treatment groups separately, the significant negative correlation remained for the PE group (*p-*value = 0.037) and not for the IVIG group (*p-*value 0.132) (Table 3).

**Table 3:** Correlation between CSF protein and duration of mechanical ventilation

	All patients	IVIG group	PE group
CSF protein (mg/dL)	149.3 ± 49.0 (110.5 to 169.0)	152.1 ± 55.7 (88.3 to 173.6)	148.4 ± 43.0 (117.5 to 166.5)
Mechanical ventilation (days)	12.0 ± 1.9 (11.0 to 13.0)	13.0 ± 2.1 (11.3 to 14.5)	11.0 ± 1.5 (11.0 to 13.0)
*p*-value	0.024^1^	0.132^1^	0.037^1^

CSF, cerebrospinal fluid; IVIG, intravenous immunoglobulin; PE, plasma exchange. Data presented as median ± SD (interquartile range)

^1^Pearson test.

There was no significant side effect attributable to any of the treatment modalities in any of the studied patients.

## Discussion

Respiratory failure among children with GBS is a serious complication requiring intensive supportive treatment in addition to the specific treatment.

The two treatment groups had no significant complications attributable to treatment intervention apart from minor hypotension episodes responding to fluid boluses in the PE group. This confirms the safety of both IVIG and PE for treating children with GBS.

The results of this study suggest that PE is more useful than IVIG as a specific treatment for the subset of children with severe rapidly progressive GBS requiring MV.

PE is believed to act by removal of circulating autoantibodies [35,36], while IVIG, among other mechanisms, is thought to work through blocking antibody production both *in vivo *[37] and *in vitro *[38,39]. Removal of autoantibodies, by PE, creates a concentration gradient between the lowered blood level and the extravascular space forcing antibody movement from the extra to the intra vascular space to be removed during the subsequent session [40]. Specific anti-ganglioside antibody levels were recently found to be associated with disease severity [41].

Children with severe, rapidly progressive GBS, as those included in this study, most likely have an intense autoantibody production with a high percentage of these antibodies already bound to nerves on development of respiratory failure. This might provide an explanation of why this subset of patients preferentially benefit from removal of already bound antibodies, PE, in comparison to blocking antibody production, IVIG.

The clinical usefulness of this relatively small difference in the duration of mechanical ventilation between PE and IVIG groups waits to be confirmed in a larger, probably multi-center, study.

CSF protein was elevated in all patients (Table 3) with no statistically significant difference between the two treatment groups (Table 1). On *post hoc *analysis, there was, however, a negative correlation between CSF protein level and duration of MV in PE but not IVIG group. This finding supports the above explanation of a shorter duration of MV in the PE group especially in view of a RCT showing CSF filtration to be at least as effective as PE for treatment of GBS [42] and the suggestion of a blood-CSF barrier dysfunction in adults and children with GBS [43].

PE group had a tendency for a shorter PICU stay, *p *= 0.094 with a comparable ability of children to walk unaided within four weeks from PICU discharge (Table 2). So, despite a shorter duration of MV, there was no difference between PE and IVIG groups regarding PICU stay or short term neurological outcome.

To our knowledge, this is the first randomized controlled study comparing PE and IVIG for treatment of children with rapidly progressive GBS requiring MV. An RCT conducted in adults with GBS showed no difference in the duration of MV in PE, IVIG and combined treatment groups [44]. This difference can be accounted for by differences in the age and PE protocol used.

It would have been more informative to perform an electrophysiological study for the study children to confirm homogeneity of the study population. It would also add to the understanding of these results to study kinetics of autoantibodies in future research with a similar design.

## Conclusions

Rapidly progressive GBS necessitating MV in children responds favorably to both IVIG and PE, with PE being superior in regard to the duration of MV but not PICU stay or short term neurological outcome. The finding of a negative correlation between CSF protein values and duration of MV in the PE group might serve as a basis for future research aiming at a better understanding of autoantibody kinetics during treatment of GBS.

## Key messages

• Children with GBS requiring MV respond favorably to both IVIG and PE.

• Using a weaning and extubation protocol, children receiving PE had a shorter duration of MV.

## Abbreviations

CSF: cerebrospinal fluid; DOI: duration of Illness; GBS: Guillain-Barré syndrome; IVIG: intravenous immunoglobulin; LP: lumbar puncture; MRC: Medical Research Council; MV: mechanical ventilation; PE: plasma exchange; PICU: pediatric intensive care unit; SBT: spontaneous breathing trial; WOB: work of breathing.

## Competing interests

The authors declare that they have no competing interests.

## Authors' contributions

MAE formulated the research idea, designed the plan, performed statistical analysis and proofread the draft. AME co-designed the plan and supervised the plasma exchange making sure of uniformity of the procedure in all studied subjects and proofread the draft. AMA followed up patients on PICU, made sure the plan was followed, and helped MAE in performing statistical analysis. ME followed up patients on PICU, drafted the manuscript, and designed statistical methodology. AAA co-formulated the research idea, co-designed the plan, followed up patients on PICU, and generated and was responsible for the random assignment of the groups. HME followed up patients on PICU, co-drafted the manuscript with ME and made sure PICU management of patients complied with the unit guidelines. All authors read and approved the final manuscript.

## Authors' information

HME is one of the founding members and the current president of the ESPIC (Egyptian Society of Pediatric Intensive Care). MAE is a member of the Board of Directors of WFPICCS (World Federation of Pediatric Intensive Care Societies) and the ambassador of WFPICCS in Egypt. An abstract of this article has been presented by MAE as an oral presentation at the 6^th ^World Congress of Pediatric Intensive Care, Sydney, 2011.
